# Euterpe Oleracea Mart. (*Açaí*) Reduces Oxidative Stress and Improves Energetic Metabolism in Myocardial Ischemia-Reperfusion Injury in Rats

**DOI:** 10.36660/abc.20180140

**Published:** 2020-01

**Authors:** Patricia Alegre, Livia Mathias, Maria Angelica Lourenço, Priscila Portugal dos Santos, Andrea Gonçalves, Ana Angélica Fernandes, Paula Schmidt Azevedo Gaiolla, Marcos Ferreira Minicucci, Leonardo Zornoff, Sergio Alberto Rupp Paiva, Bertha Furlan Polegato

**Affiliations:** 1 Universidade Estadual Paulista Júlio de Mesquita Filho - UNESP, São Paulo, SP - Brazil

**Keywords:** Euterpe Olerácea, Oxidative Stress, Energy Metabolism, Chemical Reactions, Myocardial Ischemia, Myocardial Reperfusion Injury, Rats

## Abstract

**Background:**

Euterpe oleracea Mart. (*açaí*) is a fruit with high antioxidant capacity and could be an adjuvant strategy to attenuate ischemia-reperfusion injury.

**Objective:**

To evaluate the influence of açaí in global ischemia-reperfusion model in rats.

**Methods:**

Wistar rats were assigned to 2 groups: Control (C: receiving standard chow; n = 9) and *Açaí* (A: receiving standard chow supplemented with 5% açaí; n = 10). After six weeks, the animals were subjected to the global ischemia-reperfusion protocol and an isolated heart study to evaluate left ventricular function. Level of significance adopted: 5%.

**Results:**

There was no difference between the groups in initial body weight, final body weight and daily feed intake. Group A presented lower lipid hydroperoxide myocardial concentration and higher catalase activity, superoxide dismutase and glutathione peroxidase than group C. We also observed increased myocardial activity of b-hydroxyacyl coenzyme-A dehydrogenase, pyruvate dehydrogenase, citrate synthase, complex I, complex II and ATP synthase in the A group as well as lower activity of the lactate dehydrogenase and phosphofructokinase enzymes. The systolic function was similar between the groups, and the A group presented poorer diastolic function than the C group. We did not observe any difference between the groups in relation to myocardial infarction area, total and phosphorylated NF-kB, total and acetylated FOXO1, SIRT1 and Nrf-2 protein expression.

**Conclusion:**

despite improving energy metabolism and attenuating oxidative stress, açai supplementation did not decrease the infarcted area or improve left ventricular function in the global ischemia-reperfusion model.

## Introduction

Although the mortality attributed to ischemic heart disease is declining in some countries, it still presents high morbidity, decreasing the quality of life and increasing healthcare spending.^1^ Cardiac ischemic events may be due to partial or total tissue ischemia, with reversible or irreversible myocardial dysfunction and cell death. Ischemic periods of more than 20 minutes cause irreversible damage of the cardiomyocytes and inability to functionally recover, even with the restoration of blood flow.^2,3^

During ischemia, to meet the myocardial energy demand, cellular ATP is generated by glycolysis, leading to intracellular pH reduction.^1,4^ In parallel, reduced ATP levels interrupt important active pumps in ionic homeostasis, which results in overloaded cytosolic Na^+^ and Ca^2+^, making cell repolarization unfeasible and leading to myocardial dysfunction. In addition, it is possible to observe elevated Ca^2+^ levels in cytosol, which activate enzymes (phospholipases, proteases, endonucleases and ATPases) associated with lipid peroxidation, production of reactive oxygen species (ROS), dysfunction of contractile proteins, and loss of cellular function.^4^

Although necessary to reverse ischemia, the restoration of blood flow may ultimately be more harmful than the ischemic process itself.^1,4,5^ During reperfusion, ischemia damages are worsened due to an additional discharge of ROS generated in the mitochondria by the restoration of oxygen flow.^5,6^ To improve myocardial protection during procedures involving reperfusion injury, attention has been focused on the research of drugs and substances that can prevent cardiac cell damage.^7^ In this context, we observe great interest in the antioxidant action of natural products such as *açaí.*^8^

*Açaí* (Euterpe oleracea Mart.) is a typical northern Brazilian fruit recently made popular for its high antioxidant capacity related to the presence of phenolic acids, flavonoids and anthocyanins.^9-12^*Açaí* pulp compounds consist of 31% flavonoids, 23% phenolic compounds, 11% lignoids and 9% anthocyanins.^13^ The main anthocyanins in *açaí* pulp are cyanidin-3-O-glucoside and cyanidin-3-O-rutinoside, which are responsible for the purple color of the fruit.^13^ Ferulic acid, p-hydroxybenzoic, gallic, protocatechuic, ellagic, vanillic, p-coumaric acids, and ellagic acid glycoside are the most abundant phenolic compounds.^13,14^

In experimental models, *açaí* supplementation reduced pulmonary^9^ and cerebral oxidative stress,^15^ reduced ROS formation in polymorphonuclear cells,^11,16^ decreased DNA damage, and presented anti-carcinogenic activity in bladder cancer.^10^ Oral *açaí* administration was able to attenuate hypertrophy and left ventricle dysfunction in rats subjected to myocardial infarction,^17^ but no studies on the effect of *açaí* in the global ischemia model or its effect on reperfusion injury were found.

The aim of our study was to evaluate the infarct area, left ventricle function, oxidative stress and the activity of enzymes involved in myocardial energy metabolism in the global ischemia-reperfusion model in rats after *açaí* supplementation.

## Method

### Study design

The experimental protocol of this study was approved by the Ethical Committee on the Use of Animals of the Botucatu Medical School (CEUA 1111/2014), and it is in accordance with the norms established by the National Council of Control of Animal Experimentation.

Twenty two-months-old male Wistar rats weighing 250-300 g were assigned to two groups: control (C; n = 10) and *açaí* (A; n = 10). Sample size was determined by convenience based on previous studies that used the same experimental model. Animals were kept in an environment with controlled temperature (23°C) and a 12-hour light-dark cycle in individual boxes to control feed intake. Group C received a standard chow, and the group A a standard one supplemented with 5% *açaí*^18^ for six weeks. After the supplementation period, all animals were anesthetized with sodium thiopental (80 mg/kg, IP ) to induce the global ischemia-reperfusion protocol, after which the heart was dissected. A sectional cut of the left ventricle was made to determine the infarct area, and the rest was stored in a freezer at -80°C for further analysis. One rat from the control group was lost due to technical problems during the ischemia-reperfusion protocol and the study was concluded with 9 rats in the control group and 10 rats in the *açaí* one.

### Preparation of the chow supplemented with açaí

Commercialized *açaí* pulp (Icefruit®) was defrosted and incorporated into crushed Nuvilab chow (Nuvital®). After homogenization, the chow was pelleted again, dried at 32°C and stored in a freezer at -20°C until the moment of use. The dose used in the study was 5%, as proposed by Fragoso et al.^18^

### Induction of global ischemia, reperfusion and evaluation of cardiac function

The rats were anesthetized with thiopental sodium (80 mg/kg, IP), heparinized (2,000 IU, IP) and subjected to positive pressure ventilation with 100% oxygen. Then, median sternotomy was performed, and the ascending aorta was cannulated to start retrograde perfusion with a modified Krebs-Henseleit solution (NaCl 115 mmol/L, KCl 5.4 mmol/L, CaCl_2_ 1.25 mmol/L; MgSO_4_ 1.2 mmol/L, NaH_2_PO_4_ 1.15 mmol/L, NaHCO_3_ 25 mmol/L, 11 mmol/L glucose, and 8 mmol/L mannitol). The hearts were transferred to a Langendorff apparatus (Model 830 Hugo Sachs Eletronik, Germany) with perfusion pressure at 75 mmHg. The nutrient solution was constantly oxygenated with a gas mixture of 95% O_2_ and 5% CO_2_, and the temperature was maintained at 37°C. A pacemaker was used to maintain controlled heart rate (250 bpm).

Left atrium was removed, and a latex balloon was inserted into the left ventricular cavity. The balloon was coupled to a pressure transducer and to a syringe, which allowed variation in the volume of the balloon. After 10 minutes of stabilization, the hearts were subjected to a 30-minute period of global ischemia followed by 30 minutes of reperfusion.^3^ Global ischemia was induced by completely stopping the flow of Krebs-Henseleit solution to the heart.

After ischemia and reperfusion periods, an evaluation of left ventricular function was performed. The volume inside the balloon was progressively increased to obtain left ventricular diastolic pressure variation of 0 to 25 mmHg. In addition, for each increase in volume to the balloon, the diastolic and systolic pressure, the maximum left ventricular pressure development rate (+dP/dt) and the maximum left ventricular pressure decrease rate (-dP/dt) were recorded. Diastolic pressure-volume curves were constructed.

### Analysis of infarcted myocardial area

A cross-sectional cut of the left ventricle (LV) was made - 5 mm from the apex, with a thickness of 2 mm - and incubated in phosphate buffer with 7.4 pH and 1% triphenyltetrazolium chloride (Sigma Aldrich) for 30 minutes at 37°C. After that, the sections were incubated in a 10% formaldehyde solution overnight. The ventricle sections were positioned between two glass slides and scanned to obtain the images.

Infarct area was measured through the ImageJ program by planimetry and expressed as the percentage of infarcted over total areas. In live cells the dye is reduced by dehydrogenases and appears with a dark red coloration. Dead cells lacking the enzymes are not stained and remain pale in color.^19^

### Analysis of antioxidant enzymes and lipid hydroperoxide

Samples of approximately 100 mg of LV tissue were homogenized in a sodium phosphate buffer (0.01 M) with a pH of 7.4 and centrifuged for 30 minutes at -4°C; the total proteins in the samples were quantified by Bradford method. The activities of glutathione peroxidase (GSH-Px), superoxide dismutase (SOD) and catalase in the cardiac tissue were determined by spectrophotometry according to previously described methods.^20,21^ Lipid hydroperoxide concentration in the cardiac tissue was measured by the oxidation of ammoniacal ferrous sulfate and determined by spectrophotometry.^22^

### Evaluation of energy metabolism

Samples of approximately 100 mg of LV tissue were homogenized in a sodium phosphate buffer (0.1 M, pH 7.0) and centrifuged. The supernatant was used to determine protein concentration and activity of the enzymes b-hydroxyacyl coenzyme A dehydrogenase, phosphofructokinase, lactate dehydrogenase, pyruvate dehydrogenase and citrate synthase.^23^ The pellet was re-suspended with sodium phosphate buffer (0.1 M) containing 250 mM sucrose and 2 mM EDTA and used to determine the activity of enzymatic complexes of the electron transport chain (complexes I, II and ATP synthase).^24^ Readings were performed on a microplate reader with controlGen5 2.0 software, and all reagents were obtained from the Sigma-Aldrich laboratory (Saint Louis, USA).

### Western blot

LV samples (80 mg) were homogenized with 1 ml of radio immune precipitation assay extraction buffer (RIPA), centrifuged, and the supernatant was collected. The protein in the samples was quantified by the Bradford method; and the samples were used to determine total and phosphorylated nuclear factor signaling pathway kB (NF-kB), sirtuin 1 (SIRT1), and forkhead box protein O1 (FOXO1) protein expression. To determine the nuclear factor erithroid 2 (Nrf-2), LV samples were extracted with Nuclear Extraction Buffer.^25^ All samples were diluted in Laemmli buffer.

Protein electrophoresis was performed at 4°C on an 8 to 10% polyacrylamide gel (Mini-Protean 3 Electrophoresis Cell System, Bio-Rad, Hercules, USA). After electrophoresis, the gels were transferred to nitrocellulose membranes (Mini Trans-Blot system, Bio-Rad, Hercules, USA) in a wet transfer system followed by blocking with 5% skim milk powder solution. The membrane was washed, and primary antibodies were added (Santa Cruz Biotechnology, Inc., Europe). After overnight incubation, the membrane was washed with basal solution, and secondary antibodies were added (Santa Cruz Biotechnology, Inc., Europe). After 2 hours, the membrane was washed again in basal solution.

Immunodetection was performed with the chemiluminescence method using the SuperSignal West Pico Chemiluminescent Substrate Kit (ThermoScientific, USA). Photo documentation by ImageQuant LAS 4000 (General Eletrikcs) was used to generate images, which were analyzed in Gel-Pro 32 (Media Cybernetics, Rockville, USA). The results obtained for the target proteins were normalized by glyceraldehyde-3-phosphate dehydrogenase (GAPDH) expression, and the same control animal was included in all electrophoreses for standardization between the experiments.

### Statistical analysis

Values obtained are presented as the means ± standard deviation (variables with normal distribution) or median and 25 and 75% quartile (variables with non-normal distribution). Normality was verified by the Kolmogorov-Smirnov test. Comparisons between the groups were performed with a non-pared Student’s *t-*test (variables with normal distribution) or Mann-Whitney test (variables with non-normal distribution). Statistical analysis was carried out by use of the SigmaStat software, considering significance level at 5% for all analyses.

## Results

### General features

The animals’ initial and final body weight did not differ between groups. Additionally, the left and right ventricles, liver and lung weights were similar between groups. Daily food intake was approximately 26 g for both groups (Table 1).

**Table 1:** Morphological variables and feed intake in rats submitted to global myocardial ischemia-reperfusion

	C (n = 9)	A (n = 10)	p-value
Initial BW (g)	274 ± 15	281 ± 11	0.274
Final BW (g)	468 ± 29	448 ± 40	0.225
LV weight (g)	1.03 ± 0.09	1.02 ± 0.07	0.934
RV weight (g)	0.30 ± 0.03	0.29 ± 0.03	0.431
Liver weight (g)	14.4 ± 1.6	12.9 ± 2.6	0.154
Lung weight (g)	1.62 ± 0.03	1.54 ± 0.18	0.359
Daily chow intake (g)	26.1 ± 2.2	26.6 ± 1.8	0.246

C: control group; A: açaí group; BW: body weight; LV: left ventricle; RV: right ventricle. Values are expressed as the means ± standard deviation; p-value: t test.

### Infarcted myocardial area

**Figure 1:**
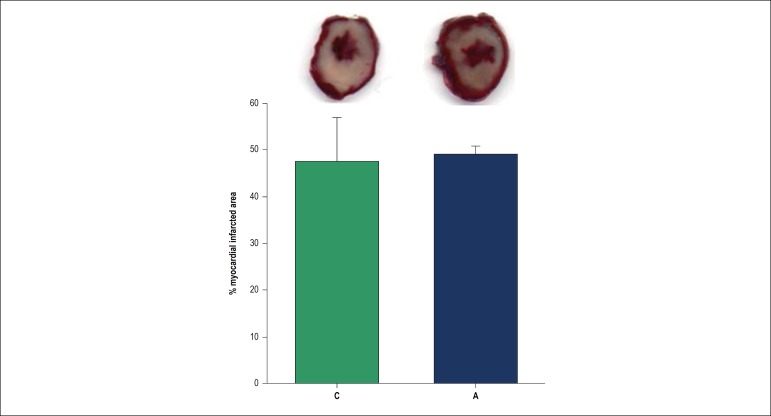
Myocardial infarcted area after global myocardial ischemia-reperfusion. Figure shows left ventricles sections stained with triphenyltetrazolium chloride 1%. White area represents the infarcted myocardium. C: control group; A: açaí group; There is no difference between groups (p = 0.710).

We observed no difference in the area of myocardial infarction between groups A and C, as observed in Figure 1.

### Oxidative stress and myocardial energy metabolism

*Açaí* supplementation promoted lower myocardial lipid hydroperoxide concentration, and greater activity of the enzymes catalase, SOD and GSH-Px in the myocardium of these rats was observed (Table 2).

**Table 2:** Myocardial oxidative stress marker and antioxidant enzymes activity after global ischemia-reperfusion

	C (n = 9)	A (n = 10)	p-value
LH (nmol/g tissue)	330 ± 33	208 ± 22	< 0.001
CAT (µmol/g of tissue)	58.5 ± 6.4	70.0 ± 8.9	0.005
SOD (nmol/mg of protein)	5.7 ± 0.4	8.1 ± 0.7	< 0.001
GSH-Px (nmol/mg of tissue)	20.2 ± 3.3	32.8 ± 4.7	< 0.001

C: control group; A: açaí group; LH: lipid hydroperoxide; CAT: catalase; SOD superoxide dismutase; GSH-Px: glutathione peroxidase. Values are expressed as the means ± standard deviation; p-value: t test.

Regarding myocardium energy metabolism, *açaí* pulp supplementation promoted lower activity of the enzymes lactate dehydrogenase and phosphofructokinase and higher activity of the enzymes b-hydroxyacyl-coenzyme A dehydrogenase, citrate synthase and pyruvate dehydrogenase. In addition, we observed higher activity of complex I, complex II and ATP synthase in the group supplemented with *açaí* (Table 3).

**Table 3:** Myocardial activity of enzymes related to energy metabolism after global ischemia-reperfusion protocol

	C (n= 9)	A (n=10)	p-value
β-hydroxyacyl-CoA dehydrogenase (nmoL/mg protein)	19.8 ± 3.3	49.8 ± 7.0	< 0.001
Phosphofructokinase (nmoL/g tissue)	181 (160 - 228)	73 (67 - 82)	< 0.001
Lactate dehydrogenase (nmoL/mg protein)	147 (145 - 167)	70 (65 - 77)	< 0.001
Pyruvate dehydrogenase (nmoL/g tissue)	114 ± 11	176 ± 19	< 0.001
Citrate synthase (nmol/mg protein)	30 (30 - 37)	100 (81 - 117)	< 0.001
Complex I (nmol/mg protein)	3.7 ± 1.0	8.0 ± 1.7	< 0.001
Complex II (nmol/mg protein)	2.1 ± 0.6	3.5 ± 0.5	< 0.001
ATP synthase (nmol/mg protein)	22.0 ± 3.9	44.5 ± 6.6	< 0.001

C: control group; A: açaí group. Values are expressed as the means ± standard deviation or median and 1st and 3rd quartile; p-value: t test or Mann-Whitney test.

### Western blot

**Figure 2:**
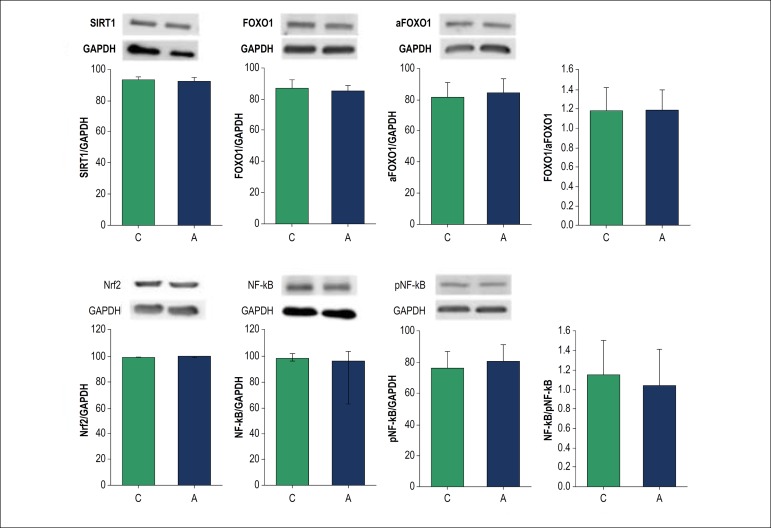
Protein expression evaluated by Western blot. C: control group; A: açaí group; SIRT1: silent information regulator 1; GAPDH: glyceraldehyde-3-phosphate dehydrogenase; FOXO1: forkhead protein 1; aFOXO1: acetylated forkhead protein 1; Nrf-2: nuclear factor erythroid 2; NF-kB: nuclear factor kappa B; pNF-kB: phosphorylated nuclear factor kappa B. All proteins expressions were normalized by GAPDH. There are no differences between groups in protein expression (p > 0.05).

The expression of the proteins involved in the regulation of the oxidative stress pathway is shown in Figure 2. There was no difference in the expression of total and phosphorylated NF-kB, total and acetylated FOXO1, SIRT1 and Nrf-2. Also, no differences were observed in the NF-kB phosphorylated/total or the FOXO1 acetylated/total ratios.

### Isolated heart study

**Figure 3:**
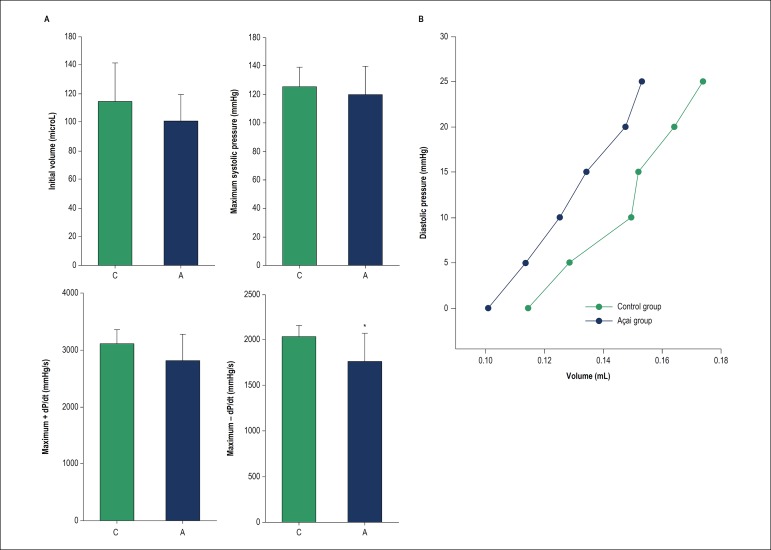
Isolated heart study after global myocardial ischemia-reperfusion. Panel A: C: control group; A: açaí group. Initial volume represents the volume inside the balloon when diastolic pressure was zero; +dP/dt: left ventricular pressure development rate; -dP/dt: left ventricular pressure decrease rate; *different from group C (p = 0.025). Panel B: Relationship between diastolic pressure and volume. The area under the curve and inclination were similar between the groups.

There was no difference between the groups regarding the initial volume of the balloon, the maximum systolic pressure reached, or +dP/dt, which represents systolic function. Group A presented worse -dP/dt than C, signifying impairment of the diastolic function in the rats that received *açaí* supplementation. The areas under the curves in the diastolic pressure-volume relationship did not differ between groups (Figure 3).

## Discussion

Myocardial ischemia-reperfusion injury is one of the leading causes of death worldwide, and it remains a situation for which current clinical therapies are surprisingly deficient.^26^ Irreversible myocardial injury progresses with increased duration of ischemia; therefore, rapid restoration of blood flow to the ischemic area is essential to save the viable myocardium.^1^ Reperfusion, however, can induce death of cardiomyocytes regardless of the ischemic episode by a process known as reperfusion injury.^1,5,27^ Decreased reperfusion injury is a key target in the battle to preserve cardiac function in patients with acute myocardial infarction.

During ischemia-reperfusion, changes such as the release of cytokines, interaction between leukocytes and endothelial cells and production of reactive nitrogen/oxygen species and free radicals (ERs)^27^ occur in cellular metabolism, which can lead to oxidative damage. ERs are generated from various sources, and the energetic metabolism, more precisely the mitochondrial electron transport chain (ETC), is one of the most important generators of these radicals.^5^

The continuous production of ERs during metabolic processes is regulated by an antioxidant defense system, which limits the intracellular levels and controls the occurrence of cellular damage. The antioxidant defense system can be enzymatic and non-enzymatic.^6,7,28^ Non-enzymatic defense system includes antioxidant compounds of dietary origin, like vitamins, mineral and phenolic compounds. Flavonoids and other compounds present in *açaí* can act as non-enzymatic antioxidant, inactivating reactive species.^29^

During ischemia, there is an increase in nicotinamide adenine dinucleotide oxidase, nitric oxide synthase, xanthine oxidase, cytochrome P450 and cyclooxygenase,^8,30^ which may result in increased ER generation; likewise, ischemia is associated with a decrease in different antioxidant enzymes. All these changes may result in oxidative stress. In this study, group A presented higher antioxidants enzymes activity in addition to lower concentration of myocardial lipid hydroperoxide. Lipid hydroperoxide is a marker of oxidative damage and is originated from the oxidative lesion of membrane lipids. Membrane lesion can lead to disturbances in permeability, alteration of the ionic flow and DNA, and impairment of extracellular matrix components.^31^

In relation to the proteins involved in the regulation of the oxidative stress pathway, the mechanisms by which phenolic dietary compounds act in the prevention of degenerative pathologies have been partially studied. The complex interactions between these dietary molecules and their molecular targets activate signaling pathways of the cellular response, including the NF-kB, Nrf2, SIRT1, and FOXO1.^32-34^

NF-kB is considered an important transcriptional factor related to oxidative stress. *Açaí* treatment inhibited NF-kB activation in astrocytes cell culture,^35,36^ inhibited NF-kB phosphorylation in microglial cells^37^ and down-regulated gene expression of NF-kB in colon myofibroblasts cell culture.^38^ Similarly, Nrf2 is an important regulator of antioxidant enzymes production. In response to oxidative stress, Nrf2 dissociates from the Keap1 protein and migrates to the cell nucleus, where it stimulates the production of antioxidant enzymes.^39^ Myocardial ischemia promotes increased protein expression of Nrf2^40^ and the effect of *açaí* on Nrf2 expression was verified in an astrocyte culture study in which the fruit reduced the protein expression of Nrf2.^41^ However, in another study, also with astrocyte culture, *açaí* administration increased Nrf2 expression,^36^ showing that the effects of *açaí* on Nrf2 are not completely understood.

Another cellular balance regulator is the SIRT1 protein, which can act on apoptosis, mitochondrial biogenesis, inflammation, glucose and lipid metabolism, autophagy and adaptations to cellular stress through the deacetylation of target proteins such as NF-kB and FOXO1. When acetylated by SIRT1, FOXO1 leads to increased expression of gluconeogenic genes.^33^ Increased transcriptional activity of FOXO1 also increases the gene expression of catalase and superoxide dismutase.^34^ To the best of our knowledge, there are no studies that evaluated the effect of açai on FOXO1 and SIRT1 protein expression.

In the normal adult heart, approximately 95% of ATP production is derived from mitochondrial oxidative phosphorylation. The adult heart normally obtains 50-70% of its ATP from fatty acid b-oxidation instead of glucose oxidation.^42^ The flavin adenine dinucleotide and nicotinamide adenine dinucleotide produced during fatty acid oxidation are used in mitochondrial ATP synthesis via oxidative phosphorylation.^43^ This is critically dependent on the maintenance of an electrochemical proton gradient across the inner mitochondrial membrane generated by the extrusion of protons from the matrix to the intermembrane space by complexes I, III and IV, which form the ETC.^44^

In our study, *açaí* supplementation led to higher activity of b-hydroxyacyl CoA-dehydrogenase and citrate synthase enzymes, which can characterize higher fatty acid oxidation. Moreover, there was lower activity of phosphofructokinase, the enzyme for glycolysis. This shows that açai supplementation altered the substrate selection for mitochondrial oxidation in reperfusion from glucose to fatty acids, maintaining energy metabolism closer to a physiological situation, as showed previously in an experimental model of renal ischemia-reperfusion.^45^ Additionally,*açaí* supplementation increased the activity of complexes I, II and ATP synthase by protecting the damage to mitochondrial complexes. However, fatty acid oxidation decreases the metabolic efficiency of injured hearts.

Interestingly, *açaí* pulp is composed predominantly by lipids that correspond to 48%.^46^ Offering more lipids could contribute to increased b-hydroxyacyl CoA-dehydrogenase activity in the treated *açaí* group. This pattern was observed in the experimental myocardial infarction model that administered a chow rich in lipids.^47^

Regarding the infarcted area, as expected for the 30-minute period of ischemia, we observed a large infarcted area in our study, approximately 50% in both groups. Previously, supplementation of a diet rich in anthocyanins in an experimental model of ischemia-reperfusion decreased the myocardial infarcted area.^48^ However, in the present study, *açaí* supplementation did not reduce infarct size.

Large infarctions usually present with important left ventricular dysfunction resulting from the impairment of cellular processes and changes in cardiac morphology. Increased oxidative stress compromises the plasma membrane permeability of myocytes, the activity of ionic pumps in the plasma membrane and in the sarcoplasmic reticulum, and intracellular calcium transit, compromising both systole and diastole.^7^ It would be expected that the attenuation of oxidative stress would be followed by an improvement in left ventricular function, as showed in different myocardial damage models.^25,49,50^ However, this study evidenced that the administration of *açaí* worsened the diastolic function after cardiac ischemia-reperfusion. This leads us to infer that the ventricular dysfunction observed in this model depends on mechanisms other than oxidative damage. Curiously, a study performed to investigate the effect of anthocyanin extract in global rat heart ischemia-reperfusion suggests that anthocyanin was cardioprotective in low doses and could be cardiotoxic in high ones.^51^

Additionally, the change in energetic metabolism that occurs in stress situations has a protective role in the myocardium.^42^ The fact that açai supplementation prevents the use of glucose and favors the maintenance of myocardial metabolism close to normal may have negatively interfered with this adaptive protective mechanism.

Whereas ischemia induces morphological and functional alterations and reperfusion injury can exacerbate these features, the discovery of new drugs, substances or strategies that can minimize cardiac damage is essential. The quantity of *açaí* that rats took was equivalent to 600 mg for a 60 kg human,^52^ which is feasible human *açaí* ingestion. Therefore, *açaí* could be a potential strategy to attenuate ischemia-reperfusion injury in clinical scenario.

However, there are some limitations that difficult the translational impact of these results. First, rats share several metabolic pathways with humans, but the organisms are quite different. Second, in this study we evaluated a global myocardial ischemia that is not the routine in clinical practice. The ischemic process in humans is the result of a long inflammatory process that can modulate completely different metabolic pathways from our model.^53^

## Conclusion

*Açaí* supplementation did not decrease the infarcted area nor improve the left ventricular function in the global ischemia-reperfusion model despite improving cardiac energy metabolism and attenuating myocardial oxidative stress, suggesting that these mechanisms may not be the main determinants of the worsened diastolic left ventricular function observed after ischemia and reperfusion with *açaí*supplementation.
